# The Phenotyping Dilemma—The Challenges of a Diversified Phenotyping Community

**DOI:** 10.3389/fpls.2019.00163

**Published:** 2019-02-28

**Authors:** Eva Rosenqvist, Dominik K. Großkinsky, Carl-Otto Ottosen, Rick van de Zedde

**Affiliations:** ^1^Department of Plant and Environmental Sciences, University of Copenhagen, Taastrup, Denmark; ^2^Department of Plant and Environmental Sciences, Copenhagen Plant Science Centre, University of Copenhagen, Frederiksberg, Denmark; ^3^Department of Food Science, Aarhus University, Aarslev, Denmark; ^4^Wageningen Plant Research, Wageningen, Netherlands

**Keywords:** phenotyping, global climate change, big data, ontology, stakeholders, breeding, omics analyses, Crop wild relatives (CWR)

In the past decade, large investments have been made for plant phenotyping in terms of funding, research hours, and high-tech installations in Europe, Australia, North America and Asia. The number of actors in phenotyping has increased rapidly and the focus has gradually shifted from basic to strategic crop research linked to classic agricultural traits. During the recent years, community-wide surveys have pinpointed focus areas, challenges, and bottlenecks in plant phenotyping (www.plant-phenotyping.org/ippn-survey_2016).

Increasing efforts addressing abiotic and biotic stresses associated with the effects of global climate change in mind are developing. Crop wild relatives (CWRs) are important sources for genes for both biotic and abiotic stress tolerance (Dempewolf et al., 2017; Vosman et al., 2018) since diversity lost during domestication is vast (Haudry et al., 2007). Within the last decade, large-scale phenotyping research platforms have been set up and are organized within national phenotyping facilities with a range of high-tech applications in climate rooms, greenhouses and in the field (e.g., www.plant-phenomics.ac.uk/, www.ipk-gatersleben.de/en/phenotyping/, www.plantphenomics.org.au).

*A more urgent challenge is however, that the phenotyping community needs to bridge the gap between academia and the multitude of stakeholders to really benefit from the huge research efforts made internationally*.

## Breeding—the Results Count, but Research can Improve the Success

**Figure 1:**
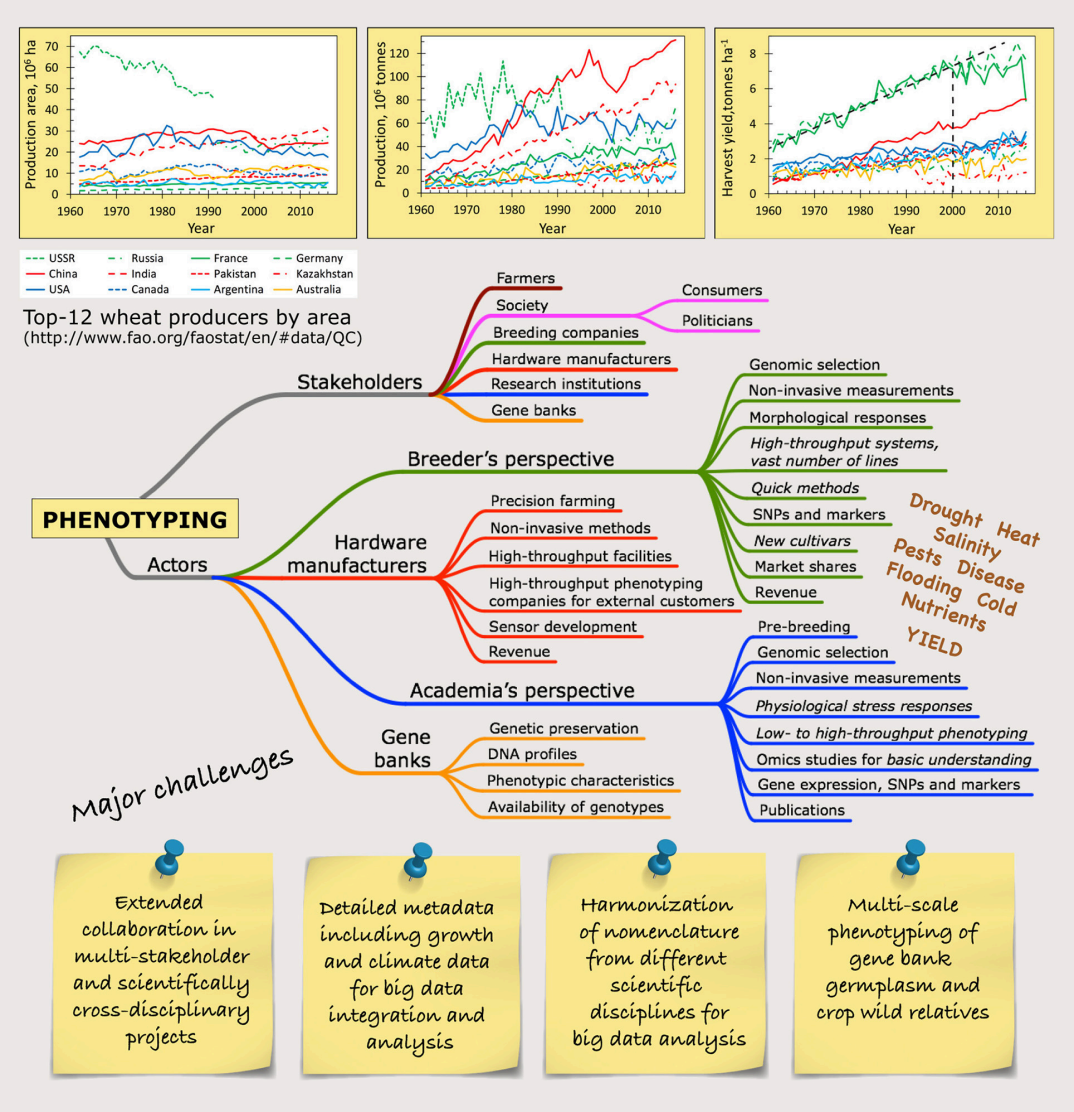
Bulletin board drawing up the current landscape of phenotyping. The development of global crop production, exemplified by the top-12 wheat producers, show that the increase in harvest yield is leveling off since year 2000 in high-yielding areas, while many big producers in terms of area and production already today suffer from environmental limitations as seen in the intermediate to low harvest yields. The mind map of the stakeholders and actors of phenotyping gives a simplified picture of the vast heterogeneity in the phenotyping community, where each focus point can be divided into all different biotic and abiotic stress factors that may be studied. Some major challenges for the years to come are posted. The lines for the top-12 wheat producers are green for Europe (USSR – narrow dashes, Russia – dash/dot, France – solid line, Germany – wide dashes), red for Asia (solid line – China, wide dashes – India, narrow dashes – Pakistan, dash/dot – Kazakhstan), dark blue for North America (solid line – USA, narrow dashes – Canada), light blue for South America (solid line – Argentina) and orange solid line for Australia.

Breeding and selection of crops have for a long time been focused on agricultural traits, disease resistance, harvest yield and quality, and to some extent stress tolerance. The yearly increase of yield in major crops is flattening (Figure 1) (Brisson et al., 2010), so new approaches are needed to change this trend (Asseng et al., 2014).

Breeders—commercial and academic—are dependent on fast and cheap evaluation tools and have until now selected cultivars primarily by evaluating the desired properties manually or by genetic markers. However, breeding is also adjusted to the different mega-environments in the world. The focus points in e.g., wheat breeding in CIMMYT (International Maize and Wheat Improvement Center) during 1945–1986 has been ca. 60% on disease resistance and ca. 40% on abiotic stress tolerance including drought and temperature resilience for cultivars aimed for different parts of the world (Ortiz et al., 2008).

The predictions of the global climate change by the Intergovernmental Panel on Climate Change (IPCC) and others indicate both increasing average temperature and CO_2_ concentration but also more extreme weather events, altogether more dynamic weather (Porter and Semenov, 2005; Porter et al., 2014). The dry regions will be drier and wet regions wetter (Dore, 2005). Model predictions even indicate that heat stress may have a greater impact on future yield than drought in Europe (Semenov and Shewry, 2011). The increased temperature will potentially decrease the yield in some areas while other will be rendered unsuitable for production (Ortiz et al., 2008).

In Europe, even small increases in temperature will have negative consequences for the agriculture in Southern Europe and positive effects in Northern Europe (van Passel et al., 2017). Many of the dominating wheat-producing countries are already today operating under environmental constrains resulting in reduced harvest yields (Figure 1), emphasizing the need for breeding for multiple stress resilience.

In addition to affecting the harvest yield, the increasing CO_2_ concentration might have a negative effect on the amount of protein in e.g., wheat (Nuttall et al., 2017) and the nutrient composition tends to be lower (Loladze, 2002; Sardans et al., 2017). The wine industry in Southern Europe will have to rely heavily on irrigation to safeguard yield (Costa et al., 2016). Even the beer production may be challenged by drought and heat in the future (Xie et al., 2018). Breeding for the future robust crops may require accepting a slightly lower but on average a more stable yield, but it requires an enormous paradigm shift to make breeders change from short-term to long-term goals.

## Defining Phenotyping

The COST (European Cooperation in Science and Technology) Action FA1306, “The quest for tolerant varieties – phenotyping at plant and cellular level” (Phenomen-All) (www.plantphenotyping.org/home_costfa1306), has worked from cell level to the field with translation to good practices for applied end use. The action revealed serious knowledge gaps within the community in handling and interpreting large data sets. Furthermore, different “languages” were detected that underline the need for harmonization of the nomenclature. It is a complex situation with a system full of legacies and a vast heterogeneity in scientific interests (Figure 1) but the more data with different standards that is accumulated in the scientific community, the harder the harmonization will be, as indicated in this web cartoon (xkcd.com/927/).

In 2017 the COST Action CA16219 Harmonious (Harmonization of UAS techniques for agricultural and natural ecosystems monitoring, www.eu/COST_Actions/ca/CA16219) was launched with the aim to harmonize measurement practices, algorithms and data processing from imaging techniques in the field. In the COST Action FA0906 UV4Growth (www.cost.eu/COST_Actions/fa/FA0906) a handbook on treatment design, measurements and plant growing conditions including minimum requirements for characterization and reporting of the growing conditions for UV-B experiments in climate chambers, greenhouses, and in the field was produced (Rosenqvist et al., 2012). The same minimum information about growth conditions is valid for phenotyping and there is a dire need for similar harmonization of other data from the numerous techniques used for phenotyping (Figure 1).

Research institutes and universities in Europe have in recent years invested in large-scale research infrastructure for automated plant phenotyping:

1. Platforms for low to high resolution, high-throughput phenomics in climate rooms and greenhouses.
2. Semi-controlled field systems for high-throughput phenomics.
3. Network of practical field experiments for lean phenotyping.

Transnational access was launched within the first EU-project European Plant Phenotyping Network (EPPN) and continued in the on-going EPPN2020 (eppn2020.plant-phenotyping.eu/) providing access to a plethora of facilities. The International Plant Phenotyping Network (IPPN) (www.plant-phenotyping.org/) was established in 2016 to connect phenotyping researchers globally. Countries in EU with the most extensive phenotyping infrastructures have initiated the project EMPHASIS, which is now on the European Strategy Forum for Research Infrastructures (EU-ESFRI) list for research infrastructures (emphasis.plant-phenotyping.eu/). EMPHASIS-PREP is the preparation phase in which six member countries define the benefit of the phenotyping community, insights and feedback are currently gathered through regional meetings and online surveys to define what services are needed.

The projects and initiatives EPPN, EPPN2020, and Phenomen-All have highlighted the demand for access and availability to phenotyping infrastructures. Phenomen-All has secured 73 early stage scientists access to research groups and their facilities by funding COST Short Term Scientific Missions. EPPN funded 65 transnational access projects at seven phenotyping installations in five countries. One of the challenges is matching the diversity of research questions to the platforms.

Results generated in climate rooms are not always directly and strongly correlated to similar experiments in the field (Spindel and McCouch, 2016). However, the ranking of the heat stress response of >1,200 wheat cultivars in climate chambers (Sharma et al., 2012) has been fully reproducible when exposing 41 selected cultivars to a milder but longer heat stress in a greenhouse (Sharma et al., 2015). The heat tolerance was characterized by the ability to sustain high values of F_v_/F_m_, photosynthesis rates and stomatal conductance and maintaining good leaf cooling throughout the heat treatment. Short heat stress in climate chambers has also been used to screen young plants of normally well-performing tomato cultivars from Nepal using chlorophyll fluorescence. The most heat tolerant and susceptible cultivars were subsequently grown in an irrigated field trial in Nepal and were by coincidence exposed to a natural heat wave (Poudyal et al., 2018). The separation into two groups in the climate chambers was fully reflected in the field. More studies like these are needed where cultivar performance after stress in protected cultivation is followed by field studies for verification of the reliability of the phenotyping methods.

Thus, to obtain a thorough understanding of the impact of climate in different regional zones on plant performance, multi-site, multi-regional experiments are needed. Furthermore, complex traits with polygenic inheritance are the ones that would put both breeders and scientists a step forward in genetic gains in breeding and ecophysiological understanding of crops (Pauli et al., 2016). To explore the mechanistic relationships needed to understand phenotyping data between non-invasive methods of specific crop traits and the underlying genetics the link has to go via multi-omics to include physiological explanations (Großkinsky et al., 2015, 2017).

The need to test the performance on a large “agricultural” scale has brought the farmers-oriented, and rapidly evolving field of precision agriculture close to the phenotyping community. Both domains require geo-referenced data linked to environmental data i.e., weather parameters, irrigation, fertilizers dosages, soil characteristics, etc. Phenotyping projects analyse these data to understand plant performance (Performance = Genetics × Environment × Management), while precision farming is focussed on the required farming activities to maximize yields.

## Stakeholders and Actors

The *stakeholders* for phenotyping range from academia in various research institutions, breeding companies, or hardware development and production, to farmers and society as a whole. The *actors* in phenotyping, though, are research institutions, breeders, hardware/software manufacturers and gene banks, supported by commercial tech companies with high-tech solutions (Figure 1). These actors have different interests and aims for their activities, which sometimes complicates collaboration.

## Public-Private Partnerships—one Solution for Collaboration

Collaborative projects with participants from breeders, seed banks, academia, and developers of phenotyping equipment are rare, but Public Private Partnerships (PPP) initiatives are funded in EU, regional, and national funding schemes, through “multi-actor” requirements. Some examples are the Nordic Plant Phenotyping Network (nordicphenotyping.org/) where a processing software for drone images has been developed for the industrial partners, and the grapevine screening in Portugal together with the University of Lisbon (Costa et al., 2016).

In the last Phenomen-All meeting in Leuven, Kristian Thorup-Kristensen presented the Danish RadiMax field infrastructure for root phenotyping, which derives from a joint project between three Danish universities and four breeding companies, where the breeders have access to most of the 600 minirhizotrons for their pipeline genotypes (Jensen, 2015). It operates down to 3 m depth and allows for manipulation of the water availability and use of labeled isotopes.

Benjamin Gillian (Crop Trust, Germany) introduced the initiative “Adapting agriculture to climate change: collecting, protecting, and preparing crop wild relatives,” which is a 10-years (2011–2020) project with core funding from the Norwegian Government with the Millennium Seed Bank in Svalbard, 21 participants and 50 other partner institutions (universities, NARS, NGO, and companies) from around the world. The project both focus on building capacity for collecting, conserving and using CWRs in developing countries and pre-breeding of wild relatives to 19 major crops creating interspecific hybrids, introgression lines and backcrosses for use in ongoing breeding programs at the same time as making the results public on www.cwrdiversity.org/.

The previous examples underlines the importance of interaction between different stakeholders to succeed in taking advantage of phenotyping.

## Big Data—Coordination and Standards

Plant phenotyping in its various approaches generates large amounts of data and the data processing is challenging. Precise ontologies, thorough experimental descriptions and sharing of data are crucial. The number of published papers on the concept “plant AND phenotyp^*^” in Web of Science has risen almost exponentially during the last 20 years from 1,002 papers in 2,000 to 4,335 in 2017. As we do not deal with really big data yet, there is still the chance to develop such data-related tools and protocols in time—but only if data pools are available and shared.

While one aspect of this challenge is the non-uniform data structures and lack of comparable standards across platforms, the more critical part is the lack of expertise in the more biologically oriented research groups interpreting the data (Krajewski et al., 2015). It is essential to implement standards for generating and describing data including a minimal amount of required metadata (Figure 1) and to make them publicly available meeting these standards to facilitate more reproducible phenotyping. In the Minimal Information about Plant Phenotyping Experiment initiative (MIAPPE, www.miappe.org/) recommendations has been developed (Cwiek-Kupczynska et al., 2016). So far, there are various commercial and academic systems of data storage of phenotyping data (cordis.europa.eu/project/rcn/95172/brief/en; Lobet et al., 2013; Arend et al., 2016; Cruz et al., 2016). Many publications do not provide the needed accessible data and accompanying metadata for further analyses. It is expected that this will gradually change with the requirement from journals and funding bodies, to store and give access to raw data for new angles of analysis. New and promising approaches to exploitation of these vast amounts of data rely on novel machine learning techniques (Tsaftaris et al., 2016; Pound et al., 2017).

Harmonization of data will be crucial in the future as it is expected that phenotypic data sets are rapidly becoming bigger and more complex. Sensor and camera systems will be more sophisticated and will be combined with complementary measurement (e.g., destructive analyses), allowing for more detailed screenings and more parameters being measured in a higher spatiotemporal resolution, i.e., more images per time and more detailed images. The data is rarely compatible between equipment or installations, which was shown in experiments in EPPN. In EPPN2020 one aim is to show the benefits of multi-site/multi-region data in comparison studies, but at least one challenge remains. Not all phenotyping production companies are willing to open their proprietary formats. In addition, established phenotypic ontologies and reliable handling of big phenotypic data could serve as a basis to make them FAIR (Findable, Accessible, Interoperable, and Re-usable), which would allow integrating them also with other information such as genetic data.

## Interaction Needed to Achieve the Goals

As the phenotyping community is extremely diverse, efficient exchange of information and open discussion of the needs of each stakeholder is needed. Christian Sig Jensen (DLF Trifolium, Denmark) introduced this aspect from a breeder's perspective in the Phenomen-All meeting in Copenhagen 2016. The breeders need methods that have a positive effect on the “breeder's equation” by increasing the breeding gains, reducing the generation interval and increasing the selection intensity and accuracy, which can be supported by automated high-throughput phenotyping approaches. However, these technologies have to be more time-efficient and/or accurate than manual breeder scorings; otherwise, they need to allow identifying novel information benefiting the breeding process. Particularly since genomic selection is implemented in breeding programs, increased phenotyping accuracy are even more important. Like the increase in publications also the number of vendors is rapidly rising, which put even more pressure on the need to document and align their systems interfaces and data standards or secure conversion tools.

## Our Joint Challenge for the Future Food Security

Recent advancement and current developments are facilitating the analysis of plants on multiple scales. Although it is a challenge regarding the amount of diverse data, it will be even more so when the irregular weather patterns of the future are becoming more obvious. These complex traits will be affected by more than one gene modification and the multi-scale will have to work on two planes; at multiple organizational levels in the plant as well as with multiple combined stresses. Phenotyping under optimal growth conditions ± drought and/or nutrient deficiency, the currently most common options for high-throughput phenotyping, will not be sufficient for major breakthroughs.

We must explore this multi-scale approach (Figure 1) which ultimately will serve basic plant science, plant breeding, and (precision) agriculture as well as collaborations between these sectors. One very important achievement of Phenomen-All became clear during the closing discussions in the last annual meeting in Leuven 2018. Even though only few formalized collaborations between academia and breeders have been initiated through the COST Action there was full consensus that the invited speakers from the breeder's community to all the Phenomen-All meetings have created a much better understanding in the European academic community now, of what breeders need in terms of phenotyping methods and produced data. It will be desirable that similar interactions between breeders, academic and other actors also improves.

## Conclusions

There is a high demand for integrated facilities where both drought and heat stress can be analyzed, generating phenotypic FAIR data, both in greenhouses and in the field. This type of collaboration requires that some “principles” of different stakeholders will have to be softened. Scientists will have to include more applied aspects in their research. Breeders will have to decrease their secrecy and open up to collaboration where pipeline cultivars are used and publications are allowed with anonymized genotypes. Hardware manufacturers will have to also develop cheap phenotyping tools and open their software storage structure to allow full access to raw data and integration of the processed data, and allow interaction between equipment from different companies.

Last but absolutely not least, a major effort is needed to develop a joint ontology within the phenotyping society to facilitate collaboration and make sure that all data comes to the best use for meta-analysis. This vast challenge is not something that will be solved by individual actors but only by a joint effort within the phenotyping society of academia and industrial stakeholders.

## Author Contributions

All authors have been part of the COST Action Phenomen-All and participated in the discussions covered by the manuscript. All authors have participated in the writing process. ER has drawn the figure.

### Conflict of Interest Statement

The authors declare that the research was conducted in the absence of any commercial or financial relationships that could be construed as a potential conflict of interest.
